# Loneliness and the onset of new mental health problems in the general population

**DOI:** 10.1007/s00127-022-02261-7

**Published:** 2022-05-18

**Authors:** Farhana Mann, Jingyi Wang, Eiluned Pearce, Ruimin Ma, Merle Schlief, Brynmor Lloyd-Evans, Sarah Ikhtabi, Sonia Johnson

**Affiliations:** 1 Division of Psychiatry, University College London, Wing B, 6th Floor, Maple House, 149 Tottenham Court Road, London, W1T 7NF UK; 2 School of Public Health Shanghai, Fudan University, Shanghai, China; 3 Department of Psychological Medicine, Kings College London, London, UK

**Keywords:** Loneliness, Social isolation, Anxiety, Public health, Depression

## Abstract

**Purpose:**

Loneliness is associated with poor health including premature mortality. There are cross-sectional associations with depression, anxiety, psychosis, and other mental health outcomes. However, it is not known whether loneliness is causally linked with the new onset of mental health problems in the general population. Longitudinal studies are key to understanding this relationship. We synthesized evidence from longitudinal studies investigating the relationship between loneliness and new onset of mental health problems, in the general population.

**Method:**

We systematically searched six electronic databases, unpublished sources, and hand-searched references, up to August 2021. We conducted a meta-analysis of eight independent cohorts and narrative synthesis of the remaining studies.

**Results:**

We included 32 studies, of which the majority focused on depression. Our narrative synthesis found most studies show loneliness at baseline which is associated with the subsequent new onset of depression. The few studies on anxiety and self-harm also showed a positive association. Our meta-analysis found a pooled adjusted odds ratio of 2.33 (95% CI 1.62–3.34) for risk of new onset depression in adults who were often lonely compared with people who were not often lonely. This should be interpreted with caution given evidence of heterogeneity.

**Conclusion:**

Loneliness is a public mental health issue. There is growing evidence it is associated with the onset of depression and other common mental health problems. Future studies should explore its impact across the age range and in more diverse populations, look beyond depression, and explore the mechanisms involved with a view to better informing appropriate interventions.

**Supplementary Information:**

The online version contains supplementary material available at 10.1007/s00127-022-02261-7.

## Introduction

Mental illness remains a leading cause of disability worldwide, and the COVID19 pandemic may mean a further increase in its burden across the population [1, 2]. Social relationships and loneliness are important candidate areas for preventive interventions, but to date, there has been no systematic review on whether loneliness in the general population is associated with new onsets of mental health problems.

Loneliness can be defined as a distressing mismatch between the quantity and/or quality of social relationships a person has, and what they desire [3]. It is related to concepts such as social capital, objective social support, and social isolation [4], but is conceptually distinct. Loneliness relates specifically to the perceived quality or quantity of social relationships, as opposed to objective assessments of them. Population surveys have suggested high levels of loneliness amongst both older and young adults (under 25), giving a roughly ‘U-shaped’ distribution [5, 6]. Those with prolonged mental health problems and/or receiving psychiatric treatment are at greatest risk of ‘severe’ loneliness (highest scores on loneliness scales). With the possibility that causality between loneliness and mental health problems could be in either direction or both, synthesizing the longitudinal evidence is an important way to clarify this.

Cross-sectional associations have recurrently been found between loneliness and several mental health problems, including depression [7], anxiety [8], personality disorder [9], psychosis [10], and suicidal ideation [11]. An important consideration has been the crossover between loneliness and depression as concepts. A number of studies have demonstrated these as partially correlated but distinct, and they may in fact share a reciprocal relationship [12].

A 2016 systematic review concluded that there is an association between poor social support and depression, but did not search for studies on loneliness, or conditions other than depression [13]. This is a significant gap as the specific subjective experience of loneliness has been demonstrated as having an important independent effect on health [14]. Of note, the review suggested that emotional support in particular (conceptually closest to loneliness) was most commonly associated with protection from depression. A more recent meta-analysis on loneliness did not include a systematic literature search, scanned the literature only for the terms ‘depression’ and ‘loneliness’, and did not look for longitudinal studies [15]. To address these gaps, and move towards establishing any causal links, we ask: ‘does loneliness lead to the onset of mental health problems in the general population?’.

## Method

### Search strategy and selection criteria

This review reports on studies of adults in the general population (i.e., non-clinical cohorts), that address the question of whether baseline loneliness is associated with the later onset of mental health problems. We included a wide range of mental health outcomes including depression, anxiety, bipolar disorder, personality disorder diagnosis, and psychosis (defined by validated tools/questionnaires and/or ICD-10/DSM criteria). In this report, our exposure of interest was ‘loneliness’. However, given that there are a number of related subjective social relationship concepts, we set out to include a broader range of terms in our original search. This was to ensure that we did not miss studies that looked at both perceived social support and loneliness as main exposures independently, but may only have listed ‘social support’ in their title/key words.

We did not include studies investigating people with intellectual disabilities, children under 16, people with organic mental illness, cohorts selected on the basis of a primary physical health diagnosis, or where loneliness and mental health problems were not the primary exposures and outcomes. The review was registered on PROSPERO (CRD42015014784).

The initial search was conducted in May 2016, as part of a broader search to include longitudinal studies on loneliness in people with established mental health problems (recently published as a separate, related review [16]). We later updated the search and this review includes studies up to and including August 2021. The databases searched were: Medline, PsychINFO, EMBASE, Web of Science, CINAHL, and the Cochrane Library. No language or publication period restrictions were applied. The reference lists of included studies were hand-searched, as well as references listed in relevant review papers. We also searched for dissertations, conference reports, or other non-published reports, on Zetoc and OpenGrey databases. Where relevant, we contacted authors for further data.

Searches were conducted using subject headings (MeSH terms) and text words within titles and abstracts. Our searches combined terms for ‘loneliness and related concepts’, ‘mental disorder’, and ‘onset’ (fuller list in Supplementary Material 1).

All identified titles were screened electronically (FM and JW for initial search, and FM, EP, MS, and SI for updates). Abstracts of relevant papers were read, and full texts retrieved if they appeared likely to meet our inclusion criteria. To assess consistency between reviewers, 20% of all titles screened by one reviewer were independently reviewed by another. All full texts of studies included by one assessor were checked by a second to ensure that they met the inclusion criteria. Any queries relating to inclusion/exclusion were resolved through discussion with a third reviewer (SJ).

### Quality assessment

We used the Mixed Methods Appraisal Tool (MMAT) Version 2011 to structure our quality assessments [17]. This is specifically designed for methodological quality appraisal across a range of study types (both quantitative and qualitative). The tool provides specific criteria against which to assess quality for each type of study. We used the quantitative, non-randomised domain designed for cohort studies. Papers are rated across the following domains: selection bias, measurement quality, adjustment for confounders, and percentage of complete outcome data/response rate/follow-up rate. Scoring the papers gives an overall rating ranging from ‘*’ to signify poorest methodological quality (one criterion met) to ‘****’ (all criteria met).

Given the focus on the general population, we included additional quality items from the Newcastle–Ottawa Scale (NOS) [18]. This was developed to assist with the evaluation of non-randomised studies, and we included two questions that ask about representativeness.

Quality assessments and data extraction findings were rated independently by reviewers (FM, JW, and MS) to assess consistency.

### Narrative and statistical analysis

We aimed to consider all included studies in a quantitative meta-analysis, but were aware it might not be possible to combine them all due to variation in statistical approach and reported results. To provide a meaningful quantitative synthesis, we pooled results from studies that provided adjusted quantifiable estimates for the risk of mental health problems. The statistical analysis was carried out using STATA v15·1, and the pooled odds ratios using random effects are reported. We measured heterogeneity between studies using the chi-squared test and the I^2^ statistic. We needed at least three independent cohorts to be eligible for meta-analysis to carry it out.

For the remaining studies, we provide a narrative synthesis, guided by the principles in Popay et al. [19]. We considered how any relationship between loneliness and mental health differed by important characteristics, such as age, gender, and study quality.

## Results

**Fig. 1:**
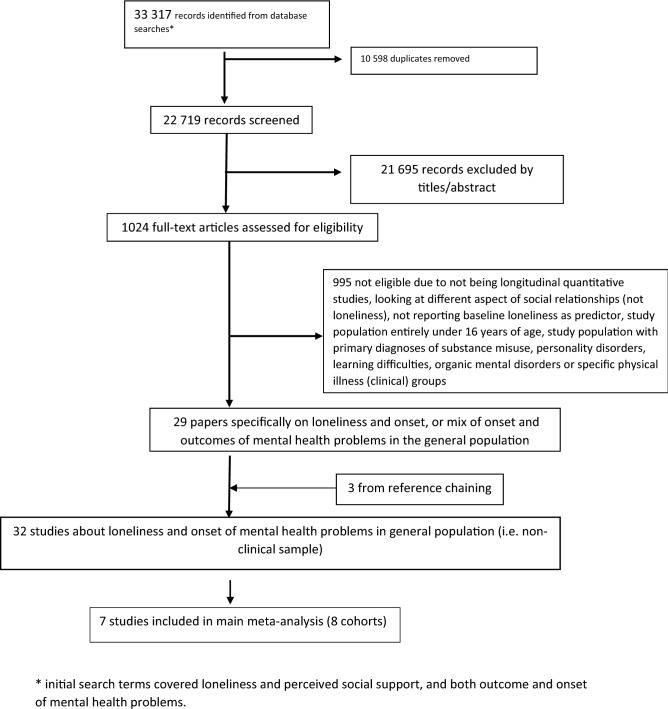
PRISMA flowchart to show search strategy. The numbers below reflect the total number of papers when combining the original search numbers with the later update. * Initial search terms covered loneliness and perceived social support, and both outcome and onset of mental health problems

The results from our search are represented in the Preferred Reporting System for Systematic Review and Meta-Analysis (PRISMA) flow diagram (Fig. 1). Our search retrieved 22,719 non-duplicate records. After screening titles and abstracts, 1024 full-text articles were assessed for eligibility. Agreement between reviewers was over 98%. Twenty-nine studies on loneliness and the onset of mental health problems, or on a combination of onset and outcomes in the general population, were included. Three studies were identified through reference chaining, giving a total of 32 studies. We contacted six authors for further data, and got responses from five.

The main characteristics and results of all 32 studies are summarized in Table 1. Overall, the majority of studies suggested baseline loneliness to be associated with the subsequent onset of depression or anxiety. There was considerable variation in sample size, ranging from 34 to 559 362 (mean 21 707). In total, accounting for studies reporting data on the same cohorts, the included papers covered 651 217 people across twenty-four countries. The length of follow-up ranged from 2 weeks to 23.5 years (mean 4.7 years). A large majority of studies (28 out of 32) had the onset of depression as a primary outcome, with five looking at both depression and anxiety [20–24], and another exclusively at anxiety disorders [25]. Two studies reported on ‘common mental health problems’ (a combination of mood and anxiety symptoms from the ‘CORE-GP’ questionnaire, or from the general health questionnaire GHQ-8 [26, 27]) and two further studies looked at suicidal ideation [28] or self-harm and suicides [29]. Over half of the studies focused on people aged over 50, with the remainder covering new mothers or younger adults, including university students. All studies of people aged under 30 were from 2017 onwards. Seven of the most recently published studies were conducted to explore loneliness and mental health in the context of the COVID19 pandemic [24, 28, 30–35]. All but two of the studies were from the US/Europe.

**Table 1:** Summary of main findings from included studies across depression, anxiety, and ‘core mental health’

Author Year Country	Quality rating^a^	Sample size and characteristics	Measures (predictor and outcome)	Length of follow-up	Results^b^ + + , + , -	Statistical analysis and main results
Depression ‘pure onset’						
Beutel (2018) Germany	***	*N* = 10,036 Age: 40–74	Loneliness 1 = no loneliness or distress; 2 = slight; 3 = moderate; 4 = severe loneliness) Depression: PHQ-9 (> 10)	5 years	+ +	Baseline loneliness associated with onset of depression at 5 years: Model 1 (excluding PHQ-9 at baseline model): loneliness aOR 2.01 (1.479, 2.71), Model 2 (including PHQ-9 at baseline, i.e. those with *subclinical* depression): loneliness aOR 1.55 (1.14, 2.10)
Conde-Sala (2019) Spain	***	*N* = 22 268 Older adults over 65	Loneliness: Hughes et al. scale Depression: 12-item EURO-D	2 years	+ +	Loneliness at baseline significantly associated with new incidence of clinically depressive symptoms at follow-up Overall across cohort: Incidence (vs no depression) aOR 1.63 (1.62, 1.64)
Green (1992) UK	*	*N* = 1070 Older people over 65	Loneliness: single item Depression: AGECAT diagnosis	3 years	+	New depression group significantly more lonely at baseline (chi-squared 16.98, *p* < 0.0005) New depression group significantly more lonely at baseline (chi-squared 16.98, *p* < 0.0005) Odds of loneliness at baseline in ‘new depression’ group significantly different (aOR 1.82)
Harris (2006) England	**	*N* = 809 Older adults over 65	Loneliness: 1-item Depression: 15-item Geriatric Depression Scale	2 years	+ +	After adjusting for confounders, loneliness at baseline predicted onset of depression; Sometimes lonely: aOR 2.8 (1.3–5.8) Often/always lonely aOR 9.3 (2.9–30.3)
Kraav 2021 (Finland)	****	*N* = 2339 Middle-aged men	Loneliness 11-item ICD-10 depression	23.5-year	+ +	Loneliness predicting new onset of depression; HR 1.04 (1.02–1.06)
Prince (1998) UK	***	*N* = 538 Older people over 65	Loneliness: single item Depression (SHORT-CARE)	1 year	+ +	Risk of new onset depression higher in people who often felt lonely RR 3.6 (2.0–6.4)
Sjoberg (2013) Sweden	***	*N* = 245 (1901 birth cohort) *N* = 310 (1930 birth cohort) Older people (recruited aged 70)	Loneliness: single item Depression: DSM-IV diagnosis	5 years	+ +	Baseline loneliness associated with onset of depression at 5 years aOR 3.81 (1.10–13.20); aOR 2.83 (1.23–6.39)
Smalbrugge (2006) Netherlands	***	*N* = 218 Older people over 55 (48% under 80)	Loneliness: De Jong Gerveld loneliness scale Depression: Geriatric Depression Scale	6 months	–	Loneliness NOT associated with onset of depression Unadjusted OR 0.07 (0.01–0.57)
Stessman (2014) Israel	**	*N* = 340 (1990 recruited cohort) *N* = 705 (1998 recruited cohort) Two cohorts: (1) Age 70–78, (2) 78–85	Loneliness: single item Depression: brief symptoms inventory	7 years	- ( +)	‘Never lonely’ vs ‘any loneliness’ NOT associated with new depression: Age 70–78 aOR 0.61 (0.137–2.68); Age 78–85 aOR 1.61 (0.8–3.25) However, categorising as ‘Never/rarely lonely’ vs ‘often or very often’ at age 78 predicts new depression at 85. aOR 2.42 (1.18–4.9)
Other depression studies						
Beller (2021) 14 European countries	**	*N* = 40 797 57% female, mean age 68	Loneliness: UCLA 3-item scale Depression: EURO-D scale	4 years	+ +	Loneliness correlated with later depression Fully adjusted *b* = 0.377 (0.297–0.456) *p* < 0.001 Stratified by gender: women B 0.480 (0.375–0.585) Men: 0.181 (0.057–0.306)
Cacioppo (2006) USA	***	Older people, aged 50–68 *N* = 212 CHASRS^c^ cohort (baseline 2002)	Loneliness: UCLA loneliness scale Depression: CES-D (minus loneliness item)	3 years	+ +	Latent growth curve modelling Baseline loneliness (year 1) predicts subsequent depression (coefficient 1.40, SE 0.55, p < 0.05)
Cacioppo (2010) USA	***	Older people, aged 50–68 *N* = 229 CHASRS^C^ cohort (baseline 2002)	Loneliness: UCLA loneliness scale Depression: CES-D (minus loneliness item)	Annual follow-up for 5 years	+ +	Significant 1-year cross-lagged effect of loneliness on depressive symptoms *B* = 0.18 (0.09–0.30) across 5 years Loneliness stable over time
Domenech-Abella (2019) Ireland	***	*N* = 5066 Community-dwelling adults aged 50 years and older	Loneliness: 5-item UCLA loneliness Depression: Composite International Diagnostic Interview-Short Form	3 waves 5–6 years	+ +	Loneliness at wave 2 predicted depression at wave 3: aOR 1.22 (1.15–1.30)
Groarke (2021) UK	***	*N* = 1958 Age range 18–87	Loneliness: UCLA 3-item Depression: PHQ-9	4 months	+ +	Loneliness at t1 associated with depression at T2 0.523 (*p* < 0.001)
Goosby (2013) USA	**	*N* = 10,564 Nationally representative sample of high school students (18 +)	Loneliness (items combined from CES-D, not validated) Depression: CES-D	2 years	+ +	Loneliness associated with new onset of depression aOR 1.41 (1.35–1.50) unadjusted OR 1.45, *p* < 0.001 Parental support across baseline and follow-up moderates the depression between loneliness and depression aOR 1.25 (1.14–1.42)
Johansson (2021) Sweden	**	*N* = 1836 University students	Loneliness: UCLA 3-item Depression: Depression and anxiety stress scale (DASS-21)	6 months	_	Loneliness association with depression at follow-ups Depression FU1: -0.79 (-1.23—0.34) FU2: -1.23(-1.71- -0.74)
Krendl (a) (2021) USA	**	*N* = 34 White female university students	Loneliness: UCLA 3-item Depression: PHQ-8	3–6 months	_	B 0.33 for loneliness predicting change in depression score, did not reach significance
Krendl (b) (2021) USA	**	*N* = 93 Older adults	Loneliness: UCLA 3-item Depression: PHQ-8	6–9 months	+ +	Greater loneliness predicted greater increases in depression score from Time 1 to Time 2 (= 0.89,.PHQ SD = 2.52), r(86) = 0.22, p = .045
Lee (2021) UK	****	*N* = 9432 Older adults	Loneliness: UCLA 3-item Depression: CES-D	12 years	+ +	For binary depression outcome: aOR univariable 1.93 (1.82–2.04); adjusted for sociodemographic factors: aOR 1.72 (1.62–1.82). fully adjusted aOR 1.28 (1.21—1.35)
Lim (2011) Singapore	*	*N* = 2799 Older people, mean age 66 (55+)	Loneliness: single item Depression: Geriatric Depression Scale Score	2 years	+ +	Loneliness significant predictor of higher depression scores after 2 years aOR 1.39, *B* = 0.33, SE 0.36, *p* = 0.03
Luoma (2015) Finland	***	*N* = 329 Mothers (recruited first trimester) Mean age 26.6	Loneliness: single item Postnatal depression (Edinburgh Postnatal Depression Score)	16 to 17-year follow-up	+ +	Group-based modelling identified a four cluster model was best for predicting depression trajectories (‘high stable’/’intermittent’/low stable’ and ‘very low’) Feeling lonely associated with high stable depression symptoms. aOR 2.1 (1.0–4.2) *p* 0.041
Okruszek (2021) Poland	**	*N* = 511 Young adults	Loneliness: UCLA-20 Depression: GHQ-30	2 weeks	_	Path analysis. Path from baseline loneliness to depression 0.05, not significant
Richardson (2017) UK	**	*N* = 454 (2 cohorts combined) University students Mean age 19.9	Loneliness: 3-item UCLA loneliness scale CES-D	2 cohorts followed up over 12–14 months	+	Baseline loneliness correlated with depression (time 2 *r* = 0.51, time 3 *r* = 0.48, time 4 *r* = 0.42) *p* < 0.001 After accounting for demographics and baseline scores, loneliness predicted depression only at T4 (*β* = 0.14, *p* < 0.05)
Theeke (2007) (thesis)	****	*N* = 13 812 Older people (50+) HRS cohort^d^	Loneliness: single item from within CES-D Depression (CES-D 7 items)	3 waves 2002–4	+	Never lonely vs chronically lonely: mean difference in depression score 1.55 (error 0.03 *p* < 0.005) Briefly lonely vs chronically lonely mean difference in depression score 0.72 (error 0.04) *p* < 0.005
Vicente (2014) Portugal	**	*N* = 83 Older people (institutionalised; average age 79.5)	Loneliness: UCLA loneliness scale Depression: GDS	2 years	–	Those whose depression scores worsened over time (including people who had no depression at baseline and people who had depression) had higher loneliness scores at baseline, but did not reach statistical significance
Luo (2012) USA	***	*N* = 2101 Older people, mean age 67 Subset of HRS cohort^d^	Loneliness: 3-item UCLA loneliness scale Depression: CES-D minus ‘I feel lonely’ and sleep items	2 years	+ +	Significant 2-year cross-lagged effect of loneliness on depression *B* = 0.132, *p* < 0.001
Xerxa (2021) USA	***	*N* = 1420 Young adult (19,21,25,39)	Loneliness: parent- reported and child-reported. Item on child and adolescent psychiatric assessment Anxiety: Young Adult Psychiatric Assessment	21 years (four time points)	+	Model adjusted for sociodemographics and childhood psychiatric problems: Childhood-reported loneliness depression aOR 1.86 (0.73–4.71) *p* 0.190; Model 2 parent-reported loneliness depression aOR 0.91 (0.32–2.57) *p* 0.861
Anxiety						
Domenech-Abella (2019) Ireland	***	*N* = 5066 Community-dwelling adults aged 50 years and older	Loneliness: 5-item UCLA loneliness scale Composite International Diagnostic Interview-Short	5–6 years	+ +	Loneliness at wave 2 predicted anxiety at wave 3: aOR 1.60 (1.10–2.34)
Flensborg-Madsen (2012) Denmark	***	*N* = 4497 Adults (mean age 44.9)	Loneliness: single item Anxiety disorder presence (ICD8/10)	13 years	+ +	Multiple Cox regression analysis Women: ‘Yes’ vs ‘no’ lonely and being hospitalized with anxiety: HR 2.01 (1.31–3.06) ‘in doubt’ vs ‘no’ HR 1.14 (0.64–2.01) Men: ‘Yes’ vs ‘no’ lonely and later being hospitalized with anxiety: HR 2.34 (1.34–4.09) ‘in doubt’ vs ‘no’ HR 2.03 (1.19–2.63)
K rendl (a) (2021)	**	*N* = 34 University students	Loneliness: UCLA 3-item Anxiety: PHQ-8	3–6 months	+	Baseline loneliness and anxiety at follow-up. beta 0.28 anxiety *p* < 0.05
Johansson (2021) Sweden	**	*N* = 1836 University students	Loneliness: UCLA 3-item Anxiety: Depression and anxiety stress scale (DASS-21)	6 months	_	Anxiety FU1 − 0.65 (− 0.98 to − 0.31) FU2 (− 0.90–1.27 to − 0.54)
Richardson (2017) UK	**	*N* = 454 (2 cohorts combined) University students Mean age 19.9	Loneliness: 3-item UCLA loneliness scale Depression: CES-DCES-D	2 cohorts followed up over 12–14 months	+	Baseline loneliness correlated with anxiety time 2 (*r* = 0.41, T3 *r* = 0.40, T4 *r* = 0.34) *p* < 0.001 After accounting for demographics, loneliness predicted anxiety only at T3 (*β* 0.15, *p* < 0.01)
Xerxa (2021) USA	***	*N* = 1420 Young adult (19,21,25,30)	Loneliness: parent- reported and child-reported. Item on child and adolescent psychiatric assessment Anxiety: Young Adult Psychiatric Assessment	21 years	+ +	Adjusted for sociodemographic factors and childhood psychiatric problems: Childhood-reported loneliness anxiety aOR 3.53 (1.55–8.04) *p* 0.002
Other mental health problems						
Ahrens (2021) Germany	****	*N* = 526 Mean age 31	Loneliness: LON 3-item, based on UCLA 3-item Mental health dysfunction: GHQ-8. ‘Significant dysfunction’ taken to be 23/24	7–8 weeks	+ +	Beta coefficient: −0.42(− 0.58 to − 0.27) *p* < 0.001 (loneliness is higher with lower scores on the version of scale used (LON)
Antonelli (2021) Brazil	**	*N* = 1674 Age range 18–75	Loneliness: UCLA 3-item Suicidal ideation (4 categories) Have you had any thoughts about killing yourself?	1 month	+ +	aOR 2.12(1.06–4.24) *p* 0.033
Nuyen (2019) Netherlands	***	*N* = 4007 General population aged 18–64 Mean age: 44.3 years	Loneliness: De Jong Gerveld loneliness scale Common mental disorders: Composite International Diagnostic Interview (CIDI) version 3.0	Baseline: 2013–2015 3 year follow-up	+ +	After adjusting for covariates, loneliness at wave 2 predicted the onset of severe 12-month CMD at wave 3; aRRR 3.28 (1–54-7.02) After adjusting for covariates, loneliness at wave 2 did not predict onset of a mild-moderate 12-month CMD; aRRR 0.94 (0.50–1.17)
Richardson (2017) UK	**	*N* = 454 (2 cohorts combined) University students Mean age 19.9	Loneliness: 3-item UCLA loneliness scale Core mental health:’ CORE-GP’	2 cohorts followed up 12–14 months	+	After adjusting for demographics and baseline scores, loneliness predicted core mental health at T2 (*β* 0.11, *p* < 0.05)
Shaw (2021) UK	****	*N* = 502 536 Middle-aged adults	Loneliness: single item: ‘do you feel lonely?” ICD-10 for suicide and self-harm	9 years	+ + (self-harm) -(suicide)	Self-harm (all *p* < 0.001) Model adjusted for ALL sociodemographic factors all physical health factors, plus perceived social support and living arrangements men: a OR 1.74 (1.40–2.76); women 1.89 (1.57–2.28 Suicide—significant univariable association (men) 3.20 (2.35–4.36), but fully adjusted model not significant 1.39 (0.97–1.99) Fully adjusted model, women 0.92 (0.54–1.57)

^a^Detailed quality ratings in supplementary material 3

^b^++ loneliness associated with onset *p* < 0.05, adjusted; + loneliness associated with onset *p* < 0.05 unadjusted,—non-significant

^c^Both drawn from same larger Chicago Health, Ageing and Social Relations study, but different statistical approaches, and follow-up

^d^Health and Retirement Study

Only one study required translation, from Portuguese to English [36]. Eleven studies took steps to screen for, and remove, people who already had mental health problems at baseline. We referred to these as ‘pure onset’ studies [25, 28, 30, 37–44].

Two papers analysed data from the Chicago Health Ageing and Retirement Study (CHASRS) cohort, but were independent studies that used different statistical approaches and reported on different follow-up periods [45, 46]. We included both sets of findings in the narrative synthesis but neither gave results that could be combined in a meta-analysis. In addition, a PhD thesis investigated loneliness in people from the Health and Retirement Study (HRS) [47] cohort, while a different study independently reported on the same cohort [48]. Again, both sets of results are described, but neither contributed to the quantitative analysis due to differences in methodology and statistical output.

The most commonly used validated loneliness measure was the UCLA Loneliness scale (both 20- and 3-item versions), used in 15 studies [49]. Three studies used the De Jong Gierveld Scale (scoring > 3 classified as ‘highly’ lonely), and the remainder used composites of relevant items in other tools (e.g., CES-D) or single items on loneliness (details in Table 1). The quality ratings of included studies ranged from the lowest ‘*’ to excellent ‘****’, with the majority being rated ‘moderate to good’. With regard to representativeness, a number of studies took steps to use, e.g., multistage probability designs to boost inclusion of ethnic minority groups, or national register data, but others were unclear on the steps in the selection process. Quality was also affected by loss to follow-up (Supplementary Material 2).

### Meta-analysis

**Fig. 2:**
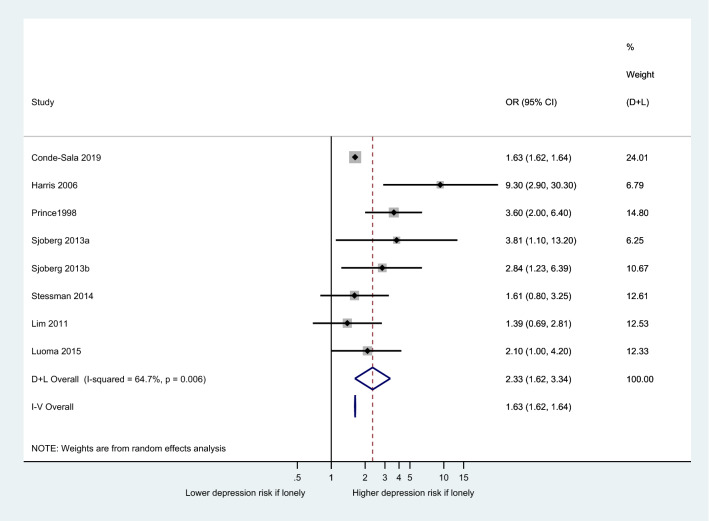
Forest plot to show association between loneliness and new onset of depression (loneliness as binary independent variable)

We performed a meta-analysis of all studies that provided adjusted odds ratios (OR) for loneliness and risk of depression. This gave a total of seven eligible studies. One study reported on two independent cohorts (1901 vs 1930 births) [38], resulting in eight distinct cohorts. A random-effects analysis was chosen given that the individual studies sampled people from distinct populations. For the result to be meaningful, we combined studies that used a binary loneliness measure (Fig. 2), but also provide a result for five studies that use continuous loneliness measures in Supplementary Material 4 .

The (adjusted) odds of depression was higher in people who were often lonely compared with those who were sometimes or never lonely. The pooled OR for loneliness being associated with subsequent onset of depression was 2.33 (1.62–3.34). This should be interpreted with a degree of caution given the *I*^2^ statistic was moderately high at 64%.

Five of the seven studies meta-analysed were ‘pure onset’ studies [37, 38, 40, 43, 50]. That is, all participants with current or past depression at baseline were excluded, so any depression at follow-up was entirely new. Six of the studies were in older adults, with the remaining one looking at new mothers. All used a range of categorical classifications of loneliness but not validated tools. The newest study [43] was the largest (*n* = 22,268). Follow-ups ranged from 6 months to 17 years, and most of the studies were of good quality (five rated ‘***’ in our quality rating). The majority of studies adjusted for gender. One study took steps to adjust for the more objective baseline measure of ‘number of contacts’[38], while others also made adjustments for objective markers such as living alone and other domains of social support [39, 40].

### Narrative synthesis

The characteristics and main results of the included studies are summarised in Table 1. Three further ‘pure onset’ depression studies all supported an association between loneliness and depression (RR 3.6 [51], aOR 1.82 [52] and HR 1.04 [44]), but did not provide results that could be meaningfully combined in the main meta-analysis. In one case [52], the lack of a confidence interval precluded its inclusion, and the original data were no longer available (author communication).

### Remaining depression studies

The studies nearly all showed a significant association between baseline loneliness and subsequent onset of depression. While there was no consistent pattern regarding study outcomes and study size, the two smallest studies did not find a significant relationship between loneliness and depression, while all seven studies with over 10,000 participants did.

Two studies [47, 48] analysed data from the HRS—a national longitudinal panel study of health and ageing in the US. Both suggested an association between loneliness and depression, but each used different combinations of items to measure loneliness and different statistical approaches (2 year cross-lagged panel analysis versus mean differences in depression score).

Both Cacioppo et al. [45] and Cacioppo et al. [46] analysed data from the CHASRS—a nationally representative US longitudinal study on health and social relationships. The 2006 study used latent growth curve modelling, and suggested baseline loneliness predicted depressive symptoms at 3 year follow-up (coefficient 1.40, SE 0.55, *p* < 0.05), while the second study reported a significant 1 year cross-lagged effect of loneliness on depression across 5 years (*B* = 0·18 (0·09–0·30)). This remained consistent when accounting for social networks, neuroticism, and demographic factors. The older study also found that depression predicted a steeper rise in loneliness levels over time, suggesting a reciprocal relationship, as did two further studies [32, 48]. Most of the studies adjusted for various social and/or clinical factors, and 11 for at least one other marker of social relationships. A 2021 study [29] of over 9000 adults that adjusted for both social support and social networks, in addition to numerous sociodemographic and clinical factors, estimated 11–18% of depression could potentially be prevented if loneliness were ‘eliminated’ (population attributable fraction).

Few studies explored mechanisms or mediators but one international study [53] noted loneliness to have a stronger effect on health outcomes in more collectivist (less individualistic) countries. However, the interaction with individualism did not reach significance in the case of depression. Another study explored whether emotional regulation was a mediating factor between loneliness and depression, but did not find a statistically significant relationship [54].

Two small studies [35, 55] did not find any statistically significant association between loneliness and depression (*n* = 34, *n* = 83). Vicente et al. found people whose loneliness scores worsened over time also had worsening depression scores (compared with people with stable or improving depression scores), but this did not reach statistical significance. A Swedish study of female undergraduates conducted during the COVID19 pandemic reported a trend in the direction of baseline loneliness predicting better mental health outcomes [33]. The study had a low response rate (27%) and the authors noted that there was very little change in depression scores overall. One study in young adults found loneliness to be associated with depression up to 8 years later [56]. Another study of young people noted that a significant association between loneliness and depression did not persist once childhood psychiatric problems were adjusted for [22]. Finally, Richardson et al. [20] found baseline loneliness was correlated with depression at all four follow-up time points. However, once baseline depression scores and demographics were adjusted for, loneliness only predicted depression at the final follow-up (12–14 months, *β* = 0·14, *p* < 0·05).

### Loneliness and anxiety

Six studies included measures of anxiety as key outcomes, and most showed a significant positive association with loneliness. Flensborg–Madsen et al. [25] followed a cohort of Danish adults over 13 years to assess onset of anxiety in that time (‘pure onset’ study). Compared with answering ‘no’ to being lonely at baseline, women responding ‘yes’ had HR 2.01 (1.31–3.05), while those responding ‘in doubt’ had HR 1.14 (0.64–2.01). In men, the corresponding HRs were 2.34 (1.34–4.09) and 2.03 (1.19–2.63). A study in young people with 21 years of follow-up [22] found that loneliness was associated with increased anxiety in adulthood, even after adjusting for several covariates including childhood psychiatric problems (aOR 3.53 (1.55–8.04) *p* 0.002). Domenech-Abella [23] found that loneliness in one wave was associated with onset of anxiety in the next (aOR 1.60, CI 1.10–2.34).

Richardson et al. [20] found baseline loneliness correlated with anxiety at 3 (*r* = 0.41), 6 (*r* = 0.40), and 12 months (*r* = 0.34) (*p* < 0.001). However, once adjusted for demographic variables, loneliness was only associated with anxiety at 6 months (β 0·15, *p* < 0·01). Finally, the Swedish study which found no significant relationship with depression [33] also reported no significant association with anxiety.

### Loneliness and other mental health problems

Antonelli et al. [28] found that loneliness was associated with suicidal ideation (aOR 2.12 (1.06–4.24), *p* 0.033), while living alone and social isolation were not, but a large British study found no significant association with the risk of death by suicide [29]. This same study did, however, report an association between loneliness and self-harm in even the most heavily adjusted model (men: aOR 1.74 (1.40–2.76), women: aOR 1.89 (1.57–2.28) both *p* < 0.001).

In a ‘pure onset’ study of common mental health ‘dysfunction’ (measured as present when the cut-off score of 23/24 on GHQ-8 was met), loneliness was associated with significant onset of mental health dysfunction over 7–8 weeks during the pandemic (− 0.42(− 0.58–0.27) *p* < 0.001—lower loneliness scores indicated greater loneliness in their measure) [30]. Richardson et al. [27] found that loneliness was associated with ‘core mental health’ at 6 months, but not at other follow-up time points. A Dutch study [57] reported on ‘common mental disorders (CMD’), classified as mild, moderate, and severe. CMD included mood disorders (including bipolar I) and substance misuse. It showed that loneliness was significantly associated with onset of ‘severe’ CMD after 12 months (aRR 3.28, 1.54–7.02), but not with mild-moderate CMD.

We found no longitudinal studies with onset of psychosis or personality disorder diagnoses as outcomes.

## Discussion

This is the first systematic review of quantitative longitudinal studies, addressing whether loneliness is associated with the subsequent onset of mental health problems. There is growing international interest in the health impacts of feeling lonely. This review focused specifically on the subjective feeling of loneliness, as opposed to a broader range of related but distinct concepts often grouped together under the umbrella of ‘social relationships’.

We established that the odds of developing new depression in adults is more than double (pooled aOR 2.33) in people who are often lonely compared with those who are not/rarely lonely. This is after adjustment for various factors. A number of studies took steps to adjust for other social relationship measures such as social support and found the effects of loneliness persisted. This is to be interpreted with a degree of caution given the heterogeneity of findings, but all included studies showed a positive association. A smaller study suggested it is possible that occasional experiences of loneliness may not in themselves lead to depressive symptoms, but more frequent or persistent loneliness might be more of a concern [58]. Understanding at which point loneliness becomes a stronger predictor of depression will be important in developing interventions.

Most of the remaining studies on depression (outside meta-analysis) also favoured loneliness being associated with an increased risk of depression onset over time. A finding of note was that three studies suggested a reciprocal relationship between depression and loneliness. While many of the studies used single item or other categorical classifications of loneliness, there was broadly no notable difference in findings between these and studies using validated loneliness measures. The choice of outcome measure did not show any pattern with regard to results either. Most studies were of ‘moderate’ to ‘good’ quality (2/3 stars out of 4) and the largest and best quality studies showed results consistent with our main conclusion that people who are lonely are at greater risk of becoming depressed.

We observed a larger number of studies in younger adults in recent years, adding to the substantial existing literature on loneliness in older people. This is important, given the peak of loneliness in young adults, as well as this being a period when mental illness can often have its onset. New mothers are a group vulnerable to loneliness as well as mental health problems, and lack of social support is broadly known to be associated with postnatal depression [59]. We observed that antenatal loneliness was associated with people scoring high for depression up to 17 years later [60]. University students are a group that have been highlighted as suffering from loneliness in the general population, both prior to and during the COVID19 pandemic [61], and our review found evidence that loneliness can lead to the onset of mental health problems in students. Studies that did not find any association between loneliness and depression highlight the need to better understand the mechanisms involved in different groups. Ensuring studies are robust enough in their methodology to draw firm conclusions, is also important.

Five out of six studies on anxiety outcomes found evidence of a positive association with loneliness. Loneliness has previously been proposed as a mediator between anxiety and depression, and is particularly associated with social anxiety in both young and older adults [62]. A more detailed understanding of how loneliness relates to developing different types of anxiety problems, and in which contexts, is needed. There was also evidence loneliness predicted the onset of common mental health problems in adults, as well as self-harm, though the number of studies was small.

Of note, there were no studies on onset of psychosis or personality disorder diagnosis identified in the search. A recent review of loneliness in people with existing psychosis noted a lack of rigorous studies [10]. Existing work in this group of people is limited to predominantly cross-sectional studies [63], partly down to the relatively lower incidence of psychotic illness.

With regards to gender, most studies that adjusted for it, or looked for interaction, did not observe any significant effects. The wider literature has not suggested a consistent picture with regard to differential effects by gender. Also of note, several of the studies took steps to adjust for other social relationship measures such as social support, and found the independent effects of loneliness persisted. Most studies did not provide subgroup analysis by ethnic origin, but some of the cohorts studied included study populations actively recruited to represent, e.g., Hispanic or Black minority groups in the US. There were no clear patterns identified in terms of ethnicity, though cross-sectional work has highlighted ethnic minority status as a risk factor for being lonely in the general population [6].

### Future research

This review has highlighted several important research gaps. There was no work on the onset of other mental health problems such as psychosis or personality disorder diagnosis, despite cross-sectional evidence loneliness is a concern in these groups [64] [8].

The trend towards more studies in younger adults in recent years is promising, though they typically sample a fairly narrow social group (university students). Given there is a peak in loneliness in young adulthood [5], which also happens to be a peak time for the onset of several mental health problems, this is an important age range to consider. There are likely to be differences in what drives loneliness, and/or how it impacts on health in people aged under 25 compared with those in their 70 s or 80 s [65]. This information will also be important in developing appropriate, effective interventions to reduce loneliness across the age range.

This review highlighted the dearth of studies beyond the US/European countries. The experiences of people in typically individualistic versus collectivist societies are an important area of inquiry. Based on our quality assessments, future studies should aim to include participants who are more broadly representative of the general population, as well as in sufficient numbers to improve follow-up rates and strengthen the accuracy of conclusions drawn.

Any association between exposure and outcome raises questions around mechanisms. There is a body of research into many different potential mechanisms through which loneliness may lead to poorer health, predominantly poorer physical health, including cardiovascular mortality. There is evidence for altered immune system function [66], changes in hypothalamic–pituitary axis function and differences in cortisol level [67], poor sleep, and altered health behaviours. It is possible some of these may be on the causal pathway (if indeed a causal association exists) between loneliness and, e.g., depression. The finding that depression in turn increases feelings of loneliness suggests a complex interplay between these experiences. Future studies of loneliness and mental health will benefit from factoring in measures of depressive symptoms, and depression has been suggested as a mediator between loneliness and poor physical health.

Cognitive mechanisms involved may also be different depending on what has led to the loneliness—physical disability versus social anxiety or bereavement, for example. There are potential cognitive, behavioural, and social factors that may be more relevant in people who are lonely versus those who are not. University students who identify as lonely tend to make more negative appraisals of how other people perceive them in actual and anticipated social circumstances [68], leading to social withdrawal and reduced informal social support. People who are more lonely also tend to engage in more ‘unhealthy’ lifestyle behaviours [69, 70]. It has been hypothesized that internalised stigma or excessive awareness of threat in social situations may be relevant in developing psychosis [71], and lonely people tend to experience these thought processes more than those who are not lonely. The experiences of people with learning disabilities or autism may also require more focused study, amongst other groups. Future research could also explore when and how loneliness becomes persistent and severe enough to cause mental health problems. A recent rapid review in children, for example suggested duration of loneliness, may be more important than intensity [72].

There is prior evidence autonomy and a sense of perceived control over one’s circumstances (in terms of loneliness/isolation) can be protective for people experiencing loneliness [73], and early studies on social support have suggested the effects on mental health can vary. Such knowledge will be important for potential strategies at alleviating loneliness.

Future policy in this area will need to consider the experiences of people across the age range, and recognise the impact loneliness has on future mental health. We have previously discussed the different levels at which interventions to target loneliness could be aimed, i.e., individual, local community, and wider society [74]. We have also discussed the importance of considering both direct and indirect interventions to address loneliness in the general population (the latter including transport, housing, and tackling poverty for example). The findings in this review further underline the importance of prioritising loneliness across these policy areas, and sit alongside existing evidence that it is associated with poor physical health outcomes.

Studies on loneliness interventions in people with established mental health problems have tended to demonstrate only modest effect sizes when they show any impact on loneliness [75], though the number of studies is small. One consideration in light of this, along with the findings from our review, is that there is also benefit intervening proactively through public mental health initiatives. Raising the profile of loneliness as a legitimate health concern is a start, and the future of interventions may include approaches including social prescribing (e.g., taking referrals from primary care) and community-level interventions [74]. Public Health England previously published estimated cost savings if loneliness in older people were to be reduced. This is likely a gross underestimate of the cost savings if loneliness were to be tackled across the age range. De Jong Gierveld has discussed the potentially superior effects of interventions that highlight a person’s need to invest in their ‘social convoys’ throughout life, before they may find themselves both unwell and lonely [76].

### Limitations

Despite its strengths, there are a number of limitations to this review. First, including children would give an even broader understanding of the life course perspective. Though beyond the scope of this particular review, the inter-relationships between the different ‘social relationship’ concepts remain an important area of research. In addition, we included studies looking at similar populations, investigating loneliness and depression. However, there were some differences in terms of the specific covariates adjusted for Table 1.

Most papers included were of moderate quality. About half used well-established validated loneliness measures. Rates of depression onset were low in some studies, which may mean an underestimation of the true effects on this outcome.

## Conclusion

This review indicates adults who experience more loneliness in the general population, are at more than twice the risk of developing depression over time. There is also evidence loneliness which is associated with increased risk of anxiety disorder, but a lack of research exploring the effects on onset of other mental health problems like psychosis. A number of important research gaps and future priorities have been identified. This review suggests that loneliness is a significant public mental health concern.

## Supplementary Information

Below is the link to the electronic supplementary material.

Supplementary file1 (DOCX 20 KB)

Supplementary file2 (DOCX 51 KB)

Supplementary file3 (DOC 116 KB)

Supplementary file4 (DOCX 18 KB)

Supplementary file5 (DOCX 19 KB)

## Data Availability

Available on request.
