# Correction to: Loneliness and the onset of new mental health problems in the general population

**DOI:** 10.1007/s00127-022-02337-4

**Published:** 2022-07-25

**Authors:** Farhana Mann, Jingyi Wang, Eiluned Pearce, Ruimin Ma, Merle Schlief, Brynmor Lloyd-Evans, Sarah Ikhtabi, Sonia Johnson

**Affiliations:** 1 Division of Psychiatry, University College London, Wing B, 6th Floor, Maple House, 149 Tottenham Court Road, London, W1T 7NF UK; 2 School of Public Health Shanghai, Fudan University, Shanghai, China; 3 Department of Psychological Medicine, Kings College London, London, UK

## Correction to: Social Psychiatry and Psychiatric Epidemiology (2022) 10.1007/s00127-022-02261-7

1. In the abstract, line 1 of method, it says '…hand-searched '*of*' references. The word 'of' should not be present. It should read: …'hand-searched references… **Language editor missed to delete “of”**
2. In the abstract, a semicolon appears in the final paragraph of the abstract (conclusion)—it should not be there. It says: 'there is evidence; …' This is not present in the version I originally submitted. **Language editor misunderstood this for two separate sentences and introduced semicolon.**
3. In the 'Methods', paragraph 4, it says 'searches were conducted subject headings…'; it should read 'searches were conducted using subject headings.' The word 'using' is missing. Please add it. **Language editor missed to insert the word “using”.**
4. In 'Results' paragraph 2, it says: 'ranging from 34 to 559 362'—for some reason there are commas appearing in the wrong parts of the number. Please correct. **This error was introduced by the technical editor by mistake.**
5. Also in 'Results', paragraph 2, it reads. …'the included papers covered 651 217 people..' but there is again a comma in the number that is not appropriate. **This error was introduced by the technical editor by mistake.**
6. pg 14, paragraph 4, 'Discussion', line 14: '…our review found *that evidence* loneliness can lead to the onset…' It should read. 'our review found *evidence that* loneliness can lead…' This is not present in the version I originally submitted. **This typo error was introduced by the Language editor. He tried to insert "that" after the word "evidence". It was done by mistake.**
7. pg 14 paragraph 4, 'Discussion', line 19 it says: 'to draw firm conclusions, which is also important…' The word 'which' should not be there. It was not in the version I submitted to SPPE. The comma before ‘is’ can be removed if you prefer it that way,and ‘which’ taken out please. **There was a comma before "is", so the Language editor inserted "which".**
8. A minor error under ‘Policy Implications’ para 1, column 2, line 9, it should say ‘sit’ and not ‘sits’.

The original article has been corrected.

