# Supplementary material for: Loneliness and the onset of new mental health problems in the general population
Source: Soc Psychiatry Psychiatr Epidemiol. 2022 May 18;57(11):2161–78. doi: 10.1007/s00127-022-02261-7 (PMC9636084; doi:10.1007/s00127-022-02261-7)
Supplement: Supplementary file 2 — Supplementary file2 (DOCX 51 KB) [file 127_2022_2261_MOESM2_ESM.docx]

| **^[[1]](#footnote-1)^Author**  **Year**  **Country** | **Quality**  **rating^a^** | **Sample size and characteristics** | **Measures (predictor and outcome)** | **Length of follow-up** | **Covariates adjusted for** | **Results^b^**  **++, +, -** | **Statistical analysis and main results** | **Comments** |
| --- | --- | --- | --- | --- | --- | --- | --- | --- |
| **Depression – ‘pure onset’ studies** | | | | | | | | |
| Beutel (2018) Germany | *** | N=10,036  Age range: 40-74; mean age 54.3 | Loneliness: single item "I am frequently alone / have few contacts" (recoded into 1 = no loneliness or distress; 2 = slight; 3 = moderate; and  4 = severe loneliness)  Depression: PHQ-9 (=/> 10) | Enrolled 2007  5-year follow-up | PHQ-9 at baseline; sociodemographic (sex, age, SES, partnership. psychological (type D, life events, social support, loneliness, GAD-2>3, social phobia, panic, history of AD), behavioural (active sports, obesity, smoking, alcohol abuse), somatic (CVD, COPD, cancer, diabetes) | ++ | Logistic regression analysis  Baseline loneliness and social support were both associated with onset of depression at 5 years:  Model 1 (excluding PHQ-9 at baseline model):  loneliness aOR 2.012 (1.479, 2.709),  social support aOR 0.926 (0.900, 0.954)  model 2 (including PHQ-9 at baseline): loneliness aOR 1.551 (1.135, 2.099)  social support aOR 0.954 (0.927, 0.984) | Excluded subjects with medical history of depression, intake of antidepressant medication, and increased depression scores (PHQ>10) at baseline |
| Conde-Sala (2019) Spain | *** | N = 31,491  Older adults over 65 | Loneliness: UCLA 3-item loneliness scale (>3/9 = loneliness)   Depression: 12-item EURO-D (≥4/12 = clinically relevant depressive symptoms) | Baseline 2013  2-year follow-up | Age, gender, schooling, financial difficulties, self-rated health, chronic diseases, ADL impairment, cognition | ++ | Multivariate binary logistic regression analyses  Loneliness at baseline significantly associated with new incidence of clinically depressive symptoms at follow-up  Loneliness associated with depression  Incidence (vs no-depression) aOR 1.63 (1.62, 1.64) Persistence (vs non-depression) aOR 3.10 (3.09, 3.11) Remission (vs persistence) OR 1.39 (1.38, 1.39) |  |
| Green  (1992)  UK | ** | N=1070  Older people over 65 | Loneliness: single item (do you feel lonely? 0=disagree 1=agree 2=strongly agree)  Depression: AGECAT diagnosis based on Geriatric Mental State data) | Enrolled 1982-3  3-year follow-up | Log-linear modelling tested independence of risk factors including age, ethnicity, alcohol use, bereavement, contact with friends or relatives, past psychiatric history | + | ‘New depression’ group was compared with those with no depression at follow-up.  New depression group significantly more lonely at baseline (chi-squared 16.98, p<0.0005)  Odds of loneliness at baseline in ‘new depression’ group significantly different (OR 1.82) | Living alone was NOT useful predictor of depression  No confidence interval available for OR (communication from author) |
| Kraav  (2021)  Finland | **** | N=2339  Middle-aged men  (mean age 53) | 11-item continuous loneliness scale  Depression: ICD-10, and Human Population Laboratory Depression Scale minus two loneliness items | Enrolled 1984-6  23.5-year follow-up | Age, year of examination, physiological changes, blood pressure, cardiovascular health | ++ | Loneliness predicting new onset of depression;  HR 1.04 (1.02-1.06). This indicates the increased hazard of getting depression with each point increase in loneliness  Also, baseline median loneliness score significantly higher in people with new onset depression (p 0.006) | Part of the Kuopio Ischaemic Heart Disease Study |
| Prince  (1998)  UK | *** | N=538  Older people over 65 | Loneliness: single item ‘often feeling lonely’  Depression (SHORT-CARE scale) | Enrolled 1993  1-year follow-up | Age, sex, five social support domains were not significant predictors of depression | ++ | Risk of new onset depression higher in people who often felt lonely RR 3.6 (2.0-6.4) | Other domains of social support were NOT significant predictors of depression. Also no significant association with age or sex. |
| Sjoberg (2013)  Sweden | *** | N=245 (1901 birth cohort)  N=310 (1930 birth cohort)  Older people (recruited aged 70), general population | Loneliness: single item (seldom/never vs sometimes/often)  Depression: DSM-IV diagnosis | 2 birth cohorts    5 years follow-up each | Sex, marital status | ++ | Baseline loneliness was associated with onset of depression at 5 years  1901 births:  aOR 3.81 (1.10-13.20)  1930 births:  aOR 2.83 (1.23-6.39) | The more objective variable ‘contact with others’ (quantity) was only associated with depression onset in older cohort |
| Smalbrugge  (2006)  Netherlands | *** | N=218  Older people over 55 (48% under 80) | Loneliness: De Jong Gerveld loneliness scale (>3/11 = ‘highly lonely’)  Depression: Geriatric Depression Scale | Enrolled 1999-2001  6 months follow-up | Age, gender, urbanisation (area), depressive symptoms, pain, functional limitations, stroke, perceived inadequacy of care | - | Loneliness NOT associated with onset of depression  Unadjusted OR 0.07 (0.01-0.57) | Very small number of people with new depression (n=10) meant adjusted OR could not be calculated  (communication from author) |
| Stessman  (2014)  Israel | ** | N=340 (1990 recruited cohort)  N=705 (1998 recruited cohort)  Two cohorts:   1. Age 70-78 2. Age 78-85 | Loneliness: single item (never lonely vs rarely/often/very often lonely)  Depression: Brief Symptoms Inventory | 2 cohorts  7 years follow-up each | Sex, marital status, education, self-rated health, physical activity, chronic pain, hypertension, ischaemic heart disease, diabetes | -  (+) | ‘Never lonely’ vs ‘any loneliness’ variable was NOT associated with new depression:  Age 70-78 aOR 0.61 (0.137-2.68)  Age 78-85 aOR 1.61 (0.8-3.25)  Categorising as ‘never/rarely lonely’ vs ‘often or very often’ at age 78 predicts new depression at 85. aOR 2.42 (1.18-4.9) | Data also analysed separately for men and women: no change to overall results. Loneliness not associated with mortality or other physical health outcomes either |
| **Depression onset and outcome** | | | | | | | | |
| Beller  (2021)  14 Europeancountries | ** | N=40 797  57% female, mean age 68 | Loneliness: UCLA 3-item scale  Depression: EURO-D scale | Study 2013-17  4-year follow-up | Sex, age, education, chronic illnesses | ++ | Fully adjusted b=0.377 (0.297-0.456) p<0.001  Stratified by gender: women B 0.480 (0.375-0.585)  Men: 0.181 (0.057-0.306)  No interaction with individism | Austria, Belgium, Switzerland, Czech Republic, Germany, Denmark, Estonia, Spain, France, Israel, Italy, Luxembourg, Sweden, Slovenia |
| Cacioppo  (2006)  USA | *** | N=212  Older people, aged 50-68  *CHASRS* cohort* | Loneliness: UCLA loneliness scale  Depression: CES-D (minus loneliness item) | Baseline 2002, 3-year follow-up | Baseline depression, stress, social support, hostility, year of study, sex, ethnicity, age, marital status, education, income | ++ | Latent growth curve modelling  Baseline loneliness (year 1) predicts subsequent depression (coefficient 1.40, SE 0.55, p<0.05) | Depression predicts loneliness as well (1.56, SE 0.7, p<0.05)  Loneliness appeared fairly stable over the three years overall  *CHASRS cohort* |
| Cacioppo  (2010)  USA | *** | N=229  Older people, aged 50-68  *CHASRS* cohort* | Loneliness: UCLA loneliness scale  Depression: CES-D  (minus loneliness item) | Annual follow-up 2002-6  4 years | Age, gender, marital status, race/ethnicity, antidepressant use, diagnosis, physical functioning | ++ | Significant 1-year cross-lagged effect of loneliness on depressive symptoms B=0.18 (0.09-0.30) across 5 years  Loneliness stable over time | Effect of loneliness was independent of demographic, health, and medication  Mix of ethnicities sampled including non-Hispanic White, Black and non-Black Latino Americans) |
| Domenech-Abella (2019)  Ireland | *** | N = 5066  Community-dwelling adults aged 50 years and older in Ireland  Mean age at baseline: 63.3 | Loneliness: 5-item UCLA loneliness scale (hardly ever or never (1) to often (2)), range from 0-10  Composite International Diagnostic Interview-Short Form (CIDI-SF) to assess MDD in past 12 months | 2009-2011 until 2014-2015; 3 waves  5-6 year-follow-up | Sociodemographic characteristics (age, sex, education, financial circumstances, widowhood, employment status, place of residence), heart diseases, somatic diseases, affective disorders, social network index (SNI) | ++ | Domenech-Abella (2019)  Ireland | *** |
| Groarke  (2021)  UK | *** | N=1958  69.8% female  Age range 18-87 (mean 37.1) | Loneliness: UCLA 3-item  Depression: PHQ-9 | Started March 23^rd^ 2020  4-month follow-up |  | ++ | Pearson’s correlation coefficient, cross-lagged structural equation modelling  Loneliness at t1 associated with depression at T2 0.523 (p<0.001) | COVID19 study |
| Goosby  (2013)  USA | ** | N=10564  Nationally representative sample of high school students (18+) | Loneliness (items combined from CES-D, not validated)  Depression: CES-D | Wave 1  1994  Wave 2  1996  2 years | Ethnicity, age, parent education, parent income, parent marital status, respondent nativity status, health insurance access, parent self-rated health, parent-reported respondent health at WAVW 1, region of residence, binge drinking frequents, regular smoking | ++ | Loneliness associated with new onset of depression  aOR 1.41 (1.35-1.50)  unadjusted OR 1.45, p<0.001  Parental support across baseline and follow-up moderates the depression between loneliness and depression aOR 1.25 (1.14-1.42) | Significant interaction between loneliness and parental support |
| Johansson  (2021)  Sweden | ** | N=1836  University students  Mean age 26.5, 73% female | Loneliness: UCLA 3-item  Depression: Depression and anxiety stress scale (DASS-21) | Started March 2020  6-month follow-up (0, 3, 6 months) | Gender, age | _ | Data not normally distributed, so used generalised estimating equations.  Depression FU1: -0.79 (-1.23—0.34)  FU2: -1.23(-1.71- -0.74) | COVID19 study |
| Krendl (a)  (2021)  USA | ** | N=34  White female university students  Mean age 19 | Loneliness: UCLA 3-item  Depression: PHQ-8 | October 2019 start  3-6-month follow-up |  | _ | Correlation coefficients:  B 0.33 for loneliness predicting change I depression score, did not reach significance | COVID19 study |
| Krendl (b)  (2021)  USA | ** | N=93  Older adults, mean age 74.7  97% white and 86% had a degree | Loneliness: UCLA 3-item  Depression: PHQ-8 | Start October 2019  6-9-month follow-up |  | ++ | correlation coefficient: change in loneliness and change in depression beta 0.15 t 1.33,  Greater loneliness between Time 2 and  Time 1 (= 0.77, SD = 2.37) predicted greater increases in depression from Time 1 to Time 2 (= 0.89,.PHQ SD = 2.52), r(86) = 0.22, p = .045. | COVID19 study |
| Lee (2021)  UK | **** | N=9432  Older adults  Mean age 65 (range 52-101) | Loneliness: UCLA 3-item  Depression: CES-D | 12-year follow-up | age, sex, marital status, education, wealth, social support, time, baseline depression, physical health, social network, BMI | ++ | univariable: 1-point increase in loneliness associated with 0.38 point increase in depression score.  0.38 (0.35-0.41) p<0.0001, adjusted for perceived social support and other social factors, and sociodemographic factors 0.33 (0.29-0.36). Fully adjusted for physical health and baseline depression 0.16 (0.13-0.19).  For binary depression outcome: aOR univariable 1.93 (1.82-2.04). adjusted for sociodemographic: aOR 1.72 (1.62-1.82). fully adjusted aOR 1.28 (1.21 - 1.35). | ELSA Study |
| Lim  (2011)  Singapore | * | N=2799  Older people, mean age 66 (55+) | Loneliness: single item dichotomised not at all lonely vs fairly lonely/very lonely  Depression: Geriatric Depression Scale Score (GDS) | 2- year follow-up | Age, gender, race, marital status, education, social contact frequency, no. of medical problems, no. of social/productive/fitness/health activities, functional disabilities, cognitive status, baseline depression and QoL | ++ | Loneliness significant predictor of higher depression scores after 2 years  aOR 1.39, B=0.33, SE 0.36, p=0.03 | Loneliness was greater contributor to model of depressive symptoms (F13.91, p<0.001 than ‘living alone’ (F1.84, P = 0.18) |
| Luo  (2012)  USA | *** | N=2101  Older people, mean age 67  *Subset of HRS cohort* | Loneliness: 3-item UCLA loneliness scale  Depression: CES-D minus ‘I feel lonely’ and sleep items | 3 waves: 2002, 2004, 2006  2-year follow-ups | Marital status, relatives/friends nearby, sleep, exercise, smoking, age, gender, ethnicity, education, household income and assets | ++ | Significant 2-year cross-lagged effect of loneliness on depression  B=0.132, p<0.001 | Reciprocal effect of depression on loneliness over two years:  B=0.113, P<0.001 |
| Luoma  (2015)  Finland | *** | N=329  Mothers (recruited first trimester, mean age 27.1)) | Loneliness: single item (always/often/sometimes vs rarely/never)  Postnatal depression (Edinburgh Postnatal Depression Score) | Baseline 1989-90  16 to 17-year follow-up | Mother’s age, past or current mental health problems, EPDS score, ever smoked, relationship changed, ‘not very good’ pregnancy, difficulties during pregnancy, negative expectations | ++ | Group-based modelling identified a four cluster model was best for predicting depression trajectories (‘high stable’/’intermittent’/low stable’ and ‘very low’)  Feeling lonely associated with high stable depression symptoms. aOR 2.1 (1.0-4.2) p 0.041 | Feeling lonely was not associated with ‘intermittent’ trajectory |
| Okruszek  (2021)  Poland | ** | N=511  Young adults, mostly students living in a large city  81% female | Loneliness: UCLA 20-item  Depression: GHQ-30 | 2-week follow-up |  | - | Path analysis. Path from baseline loneliness to depression 0.05, not significant | COVID19 study |
| Richardson  (2017)  UK | ** | N=454 (2 cohorts combined)  University students  Mean age 19.9 | Loneliness: 3-item UCLA loneliness scale  CES-D | 2012-2014; 2 cohorts followed up over 12-14 months  First cohort (baseline Feb-June 2012): FU at 3/12, 6/12 and 12/12  Second cohort (baseline Oct/Dec 2012): FU at 3/12 and 6/12, 12/12 | Age, gender, ethnicity, baseline scores | + | Baseline loneliness correlated with depression (time 2 r=0.51, time 3 r=0.48, time 4 r=0.42) p<0.001  After accounting for demographics and baseline scores, loneliness predicted depression only at T4 (beta=0.14, p<0.05) | No evidence that presence of mental health problems predicted increased loneliness over time  Sample nearly 80% female |
| Theeke  (2007)  (thesis)  USA | *** | N= 13 812  Older people (50+)  *HRS cohort* | Loneliness: single item from within CES-D (‘feeling lonely for most of past week). ‘Never lonely’ vs ‘briefly lonely’ vs ’chronically lonely’  Depression (CES-D 7 items) | Waves 2002-4  2 years | Independent analysis of covariance tests to control for marital status, health, education, functional statues, chronic illness, age, income, no. of people in household | + | Never lonely vs chronically lonely: mean difference in depression score 1.55 (error 0.03 p<0.005)  Briefly lonely vs chronically lonely mean difference in depression score 0.72 (error 0.04) p<0.005 | Analysis of covariance showed results for loneliness on depression remained significant |
| Vicente  (2014)  Portugal | ** | N=83  Older people (institutionalised; mean age 79.5) | Loneliness: UCLA loneliness scale  Depression: GDS | 2011-2013  2-year follow-up |  | - | Those whose depression scores worsened over time (including people who had no depression at baseline and people who had depression) had higher loneliness scores at baseline, but did not reach statistical significance | Of those whose loneliness scores worsened over time, a significantly greater proportion had worsening depression scores (compared with people whose depression scores improved or they remained depression-free). |
| Xerxa  (2021)  USA | *** | N= 1420  Predominantly rural, 49% female  Outcomes at age 19, 21, 25,39 | Loneliness: parent- reported and child-reported. Item on child and adolescent psychiatric assessment.  Anxiety: Young Adult Psychiatric Assessment | Began January 1993; recruited aged 9  21-year follow-up in total. From age 19 at four timepoints. | sex of the child, rural vs urban , family hardships (including low socio-economic status, single parent, change in parent structure, maltreatment, & depression of mother), childhood psychiatric comorbidities. | + | Child-reported loneliness:, depression aOR 2.72 (1.08-6.83). Parent-reported loneliness depression aOR 1.61 (0.61-4.23) 0.331. Model 2 ADDITIONALLY adjusted for childhood psychiatric problems: Childhood-reported loneliness depression aOR 1.86 (0.73-4.71) p 0.190 ; Model 2 parent-reported loneliness depression aOR 0.91 (0.32-2.57) p 0.861 They also looked at loneliness trajectories. MODEL 1: childhood-reported (high vs low loneliness trajectory) depression aOR 3.71 (1.09-7.01) p<0.0001; BETA COEFFICIENTS p<0.0001, depression B 2.34 (0.70-1.82) p<0.0001. MODEL 2: depression B 1.68 (0.21-1.41) p 0.001 | No significant association with depression when full model adjusted for childhood psychiatric problems |
| **Anxiety** | | | | | | | | |
| Domenech-Abella (2019)  Ireland | **** | N = 5066  Community-dwelling adults aged 50 years and older in Ireland  Mean age at baseline: 63.3 | Loneliness: 5-item UCLA loneliness scale (hardly ever or never (1) to often (2)), range from 0-10  Composite International Diagnostic Interview-Short Form (CIDI-SF) to assess GAD lasting six months or longer | 2009-2011 until 2014-2015; 3 waves  5-6 year-follow-up | Sociodemographic characteristics (age, sex, education, financial circumstances, widowhood, employment status, place of residence), heart diseases, somatic diseases, affective disorders, social network index (SNI) | ++ | After adjusting for covariates, loneliness at wave 2 predicted anxiety at wave 3: aOR 1.60 (1.10-2.34) |  |
| Flensborg-Madsen  (2012)  Denmark | *** | N=4497  Adults (mean age 44.9) | Loneliness: single item (no, in doubt, yes)  Anxiety disorder presence (national registers ICD8: 300, 300.2, 300.3, ICD-10: F40-43) | Enrolled 1993  13-year-follow-up | Age, yearly income, number of diseases at times of investigation | ++ | Multiple Cox regression analysis  Women: ‘Yes’ vs ‘no’ lonely and being hospitalized with anxiety: HR 2.01 (1.31-3.06)  ‘in doubt’ vs ‘no’ HR 1.14 (0.64-2.01)  Men: ‘Yes’ vs ‘no’ lonely and later being hospitalized with anxiety: HR 2.34 (1.34-4.09)  ‘in doubt’ vs ‘no’ HR 2.03 (1.19-2.63) |  |
| Krendl (a)  (2021)  USA | ** | N=34  White female university students  Mean age 19 | Loneliness: UCLA 3-item  Anxiety: PHQ-8 | October 2019 start  3-6-month follow-up |  | _ | Correlation coefficients:  Baseline loneliness and anxiety at follow-up. beta 0.28 anxiety p<0.05 | COVID19 study |
| Johansson  (2021)  Sweden | ** | N=1836  University students  Mean age 26.5, 73% female | Loneliness: UCLA 3-item  Anxiety: Depression and anxiety stress scale (DASS-21) | Started March 2020  6-month follow-up (0, 3, 6 months) | Gender, age | _ | Data not normally distributed, so used generalised estimating equations.  Anxiety FU1 -0.65 (-0.98 to -0.31) FU2 (-0.90-1.27 to -0.54) | COVID19 study |
| Richardson  (2017)  UK | ** | N=454 (2 cohorts combined)  University students  Mean age 19.9 | Loneliness: 3-item UCLA loneliness scale  CES-D | 2012-2014; 2 cohorts followed up over 12-14 months  First cohort (baseline Feb-June 2012): FU at 3/12, 6/12 and 12/12  Second cohort (baseline Oct/Dec 2012): FU at 3/12 and 6/12, 12/12 | Age, gender, ethnicity, baseline scores | + | Baseline loneliness correlated with anxiety time 2 (r=0.41, T3 r=0.40, T4 r=0.34) p<0.001  After accounting for demographics, loneliness predicted anxiety only at T3 (beta 0.15, p<0.01) | Study looked at depression , anxiety, and ‘core mental health’ (below) |
| Xerxa  (2021)  USA | *** | N= 1420  Predominantly rural, 49% female  Outcomes at age 19, 21, 25,39 | Loneliness: parent- reported and child-reported. Item on child and adolescent psychiatric assessment.  Depression: Young Adult Psychiatric Assessment | Began January 1993; recruited aged 9  21-year follow-up in total. From age 19 at four timepoints. | sex of the child, rural vs urban , family hardships (including low socio-economic status, single parent, change in parent structure, maltreatment, & depression of mother), childhood psychiatric comorbidities. | ++ | Child-reported loneliness: anxiety aOR 5.97 (2.77-12.7) p<0.0001. Parent-reported loneliness anxiety aOR 2.95 (1.51-5.75) p<0.0001,. Model 2 ADDITIONALLY adjusted for childhood psychiatric problems: Childhood-reported loneliness anxiety aOR 3.53 (1.55-8.04) p 0.002, d; Model 2 parent-reported loneliness anxiety aOR 2.95 (1.51-5.75) p 0.001 They also looked at loneliness trajectories. MODEL 1: childhood-reported anxiety (high vs low loneliness trajectory) aOR 6.34 (2.78-9.94) p<0.0001, BETA COEFFICIENTS anxiety B 2.65 (1.21-2.76) p<0.0001, MODEL 2: anxiety B 2.31 (0.55-2.05) p 0.001, | Measured both depression and anxiety |
| **Other** |  |  |  |  |  |  |  |  |
| Ahrens  (2021)  Germany | **** | N=526  68.6% female, | Loneliness: LON 3-item, based on UCLA 3-item  Mental health dysfunction: GHQ-8. ‘Significant dysfunction’ taken to be 23/24, based on previous literature. | Started 31^st^ March 2020  Weekly questionnaire  7-8 weeks | perceived social support, changes in social support, stress, daily hassles from COVID19, worries about COVID19, critical events, cognitive emotional regulation | ++ | Beta coefficient:  -0.42(-0.58- -0.27) p<0.001 (loneliness is higher with lower scores on the version of scale used (LON) | ‘pure onset’ study  Subsample of ‘LORA’ cohort |
| Antonelli  (2021)  Brazil | ** | N=1674  Age range 18-75, mean age 38.6  86.5% female | Loneliness: UCLA 3-item  Suicidal ideation (4 categories) Have you had any thoughts about killing yourself? | 1-month follow-up | age, gender, sexual orientation, marital status, income, geographical area, education, healthcare professional, unemployment, financial crisis during the pandemic, family relationship quality, religion, meditation, sleep quality, physical activity, childhood trauma, previous suicide attempts, family history of suicide, PHQ-9, GAD-7, AUDIT-C, cocaine and benzodiazepine use | ++ | aOR 2.12(1.06-4.24) p 0.033 | ‘pure onset’ study |
| Nuyen (2019)  Netherlands | *** | N=4007  General population aged 18-64  Mean age: 44.3 years | Loneliness: De Jong Gierveld loneliness scale  Common mental disorders: Composite International Diagnostic Interview (CIDI) version 3.0 | Baseline: 2013-2015  3-year follow-up | gender, age, education, living situation, job status, household income, recent negative life event, perceived social support | ++ | After adjusting for covariates, loneliness at wave 2 predicted the onset of severe 12-month CMD at wave 3;  aRRR 3.28 (1-54-7.02)  After adjusting for covariates, loneliness at wave 2 did not predict onset of a mild-moderate 12-month CMD;  aRRR 0.94 (0.50-1.17) |  |
| Richardson  (2017)  UK | ** | N=454 (2 cohorts combined)  University students  Mean age 19.9 | Loneliness: 3-item UCLA loneliness scale  Core mental health:’ CORE-GP’ | 2012-2014; 2 cohorts followed up over 12-14 months  First cohort (baseline Feb-June 2012): FU at 3/12, 6/12 and 12/12  Second cohort (baseline Oct/Dec 2012): FU at 3/12 and 6/12, 12/12 | Age, gender, ethnicity, baseline scores | + | After adjusting for demographics and baseline scores, loneliness predicted core mental health at T2 (beta 0.11, p<0.05). |  |
| Shaw  (2021)  UK | **** | N= 502 536  Middle-aged adults  Age range 37-73 | Loneliness: single item: ‘do you feel lonely?”  ICD-10 for suicide and self-harm | 9-year follow-up | age, sex, ethnicity, employment, education, deprivation, same sex partner, physical morbidities, past depression, medication, alcohol, smoking | ++ (self-harm)  -  (suicide) | SELF-HARM RESULTS (all p<0.001): univariable HR for loneliness, men: 5.32(4.43-6.40), several models adjusted for increasing number of covariates. Model adjusted for ALL sociodemographic factors men: 2.01 (1.63-1.47), model adjusted for all sociodemographic factors, all physical health factors, plus perceived social support and living arrangements men: 1.74 (1.40-2.76)  Women: univariable 4.35 (3.65-5.13), adjusted for all sociodemographic factors 1.92 (1.61-2.29); adjusted for sociodemographic, physical health and perceived social support 1.89 (1.57-2.28).  SUICIDE: Men univariable 3.20(2.35-4.36) p<0.001, adjusted for sociodemographics 2.45 (1.77-3.40) p<0.001, adjusted fully 1.39 (0.97-1.99) Women univariable 1.49(0.91-2.43), adjusted for sociodemographic factors 0.92(0.55-1.54), adjusted for demographic and physical health/perceived social support 0.92 (0.54-1.57) – not significant | Biobank study |

a Detailed quality ratings in Supplementary Material 2

b ++ p<0.05, adjusted ; + p<0.05 unadjusted, - non-significant

c both drawn from same larger Chicago Health, Aging and Social Relations study, but different statistical approaches, and follow-up, d Health and Retirement Study

**Supplementary material Table 1 Characteristics and main findings from included studies across depression, anxiety and other outcomes**

Loneliness and the onset of new mental health problems in the general population

Social Psychiatry and Psychiatric Epidemiology

Farhana Mann*, Jingyi Wang, Eiluned Pearce, Ruimin Ma, Merle Schleif, Brynmor Lloyd-Evans, Sarah Ikhtabi, Sonia Johnson

*Division of Psychiatry, University College London Email: farhana.mann@ucl.ac.uk

1. [↑](#footnote-ref-1)
