# Supplementary material for: Loneliness and the onset of new mental health problems in the general population
Source: Soc Psychiatry Psychiatr Epidemiol. 2022 May 18;57(11):2161–78. doi: 10.1007/s00127-022-02261-7 (PMC9636084; doi:10.1007/s00127-022-02261-7)
Supplement: Supplementary file 4 — Supplementary file4 (DOCX 18 KB) [file 127_2022_2261_MOESM4_ESM.docx]

Supplementary Figure 4

Forest Plot to show association between baseline loneliness and onset of depression (continuous loneliness measures).

Loneliness and the onset of new mental health problems in the general population

*Social Psychiatry and Psychiatric Epidemiology*

Farhana Mann*, Jingyi Wang, Eiluned Pearce, Ruimin Ma, Merle Schleif, Brynmor Lloyd-Evans, Sarah Ikhtabi, Sonia Johnson

*Division of Psychiatry, University College London Email: farhana.mann@ucl.ac.uk
